# Exploration in the Mechanism of Ginsenoside Rg5 for the Treatment of Osteosarcoma by Network Pharmacology and Molecular Docking

**DOI:** 10.1111/os.13971

**Published:** 2023-12-12

**Authors:** Ming‐yang Liu, Dong‐xin Jiang, Xiang Zhao, Liang Zhang, Yu Zhang, Zhen‐dong Liu, Run‐ze Liu, Hai‐jun Li, Xiao‐yu Rong, Yan‐zheng Gao

**Affiliations:** ^1^ Henan Province Intelligent Orthopedic Technology Innovation and Transformation International Joint Laboratory, Henan Key Laboratory for Intelligent Precision Orthopedics, Department of Surgery of Spine and Spinal Cord, Henan Provincial People's Hospital, People's Hospital of Zhengzhou University People's Hospital of Henan University Zhengzhou China; ^2^ Department of Immunity, Institute of Translational Medicine The First Hospital of Jilin University Jilin China

**Keywords:** Ginsenoside Rg5, Molecular docking, Network pharmacology, Osteosarcoma, Survival analysis

## Abstract

**Objective:**

Osteosarcoma is a primary malignancy originating from mesenchymal tissue characterized by rapid growth, early metastasis and poor prognosis. Ginsenoside Rg5 (G‐Rg5) is a minor ginsenoside extracted from *Panax ginseng* C.A. Meyer which has been discovered to possess anti‐tumor properties. The objective of current study was to explore the mechanism of G‐Rg5 in the treatment of osteosarcoma by network pharmacology and molecular docking technology.

**Methods:**

Pharmmapper, SwissTargetPrediction and similarity ensemble approach databases were used to obtain the pharmacological targets of G‐Rg5. Related genes of osteosarcoma were searched for in the GeneCards, OMIM and DrugBank databases. The targets of G‐Rg5 and the related genes of osteosarcoma were intersected to obtain the potential target genes of G‐Rg5 in the treatment of osteosarccoma. The STRING database and Cytoscape 3.8.2 software were used to construct the protein–protein interaction (PPI) network, and the Database for Annotation, Visualization and Integrated Discovery (DAVID) platform was used to perform gene ontology (GO) and Kyoto Encyclopedia of Genes and Genomes (KEGG) pathway enrichment analyses. AutoDock vina software was used to perform molecular docking between G‐Rg5 and hub targets. The hub genes were imported into the Kaplan–Meier Plotter online database for survival analysis.

**Results:**

A total of 61 overlapping targets were obtained. The related signaling pathways mainly included PI3K‐Akt signaling pathway, Proteoglycans in cancer, Lipid and atherosclerosis and Kaposi sarcoma‐associated herpesvirus infection. Six hub targets including PIK3CA, SRC, TP53, MAPK1, EGFR, and VEGFA were obtained through PPI network and targets‐pathways network analyses. The results of molecular docking showed that the binding energies were all less than –7 kcal/mol. And the results of survival analysis showed TP53 and VEGFA affect the prognosis of sarcoma patients.

**Conclusion:**

This study explored the possible mechanism of G‐Rg5 in the treatment of osteosarcoma using network pharmacology method, suggesting that G‐Rg5 has the characteristics of multi‐targets and multi‐pathways in the treatment of osteosarcoma, which lays a foundation for the follow‐up experimental and clinical researches on the therapeutic effects of G‐Rg5 on osteosarcoma.

## Graphical abstract

The pharmacological targets of Rg5 were selected from the Pharmmapper, SwissTargetPrediction and similarity ensemble approach databases. The osteosarcoma genes were found from the GeneCards, OMIM, and drugbank databases.The targets of G‐Rg5 and the related genes of osteosarcoma were intersected to obtain the potential target genes of G‐Rg5 against osteosarcoma. The STRING database and Cytoscape 3.8.2 software were used to construct the protein–protein interaction (PPI) network. Gene ontology (GO) and Kyoto Encyclopedia of Genes and Genomes (KEGG) pathway enrichment analyses were identified by the DAVID database. Hub genes were obtained through PPI network and targets‐pathways network analyses. AutoDock vina software was used to perform molecular docking between G‐Rg5 and hub targets. The hub genes were then imported into the Kaplan–Meier Plotter online database for survival analysis.

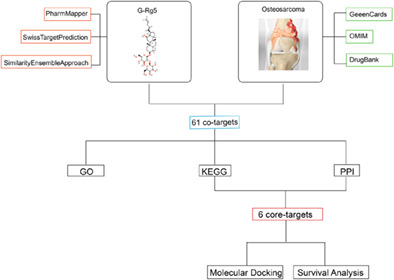

## Introduction

Osteosarcoma is a primary malignant tumor originating from mesenchymal tissue, which is characterized by high malignancy, rapid growth, early metastasis and poor prognosis.^1^, ^2^ It usually occurs in adolescents or elderly patients.^3^ Most patients undergo surgery combined with new‐adjuvant chemotherapy that include preoperative chemotherapy, surgical resection, and postoperative chemotherapy. Although the 5‐year survival rate has improved, the prognosis is still not optimistic.^2^, ^4^ Moreover, chemotherapy drugs have large side effects and are easy to develop chemoresistance.^5^ Therefore, there is an urgent need for new drug candidates on osteosarcoma treatment.

**FIGURE 1:**
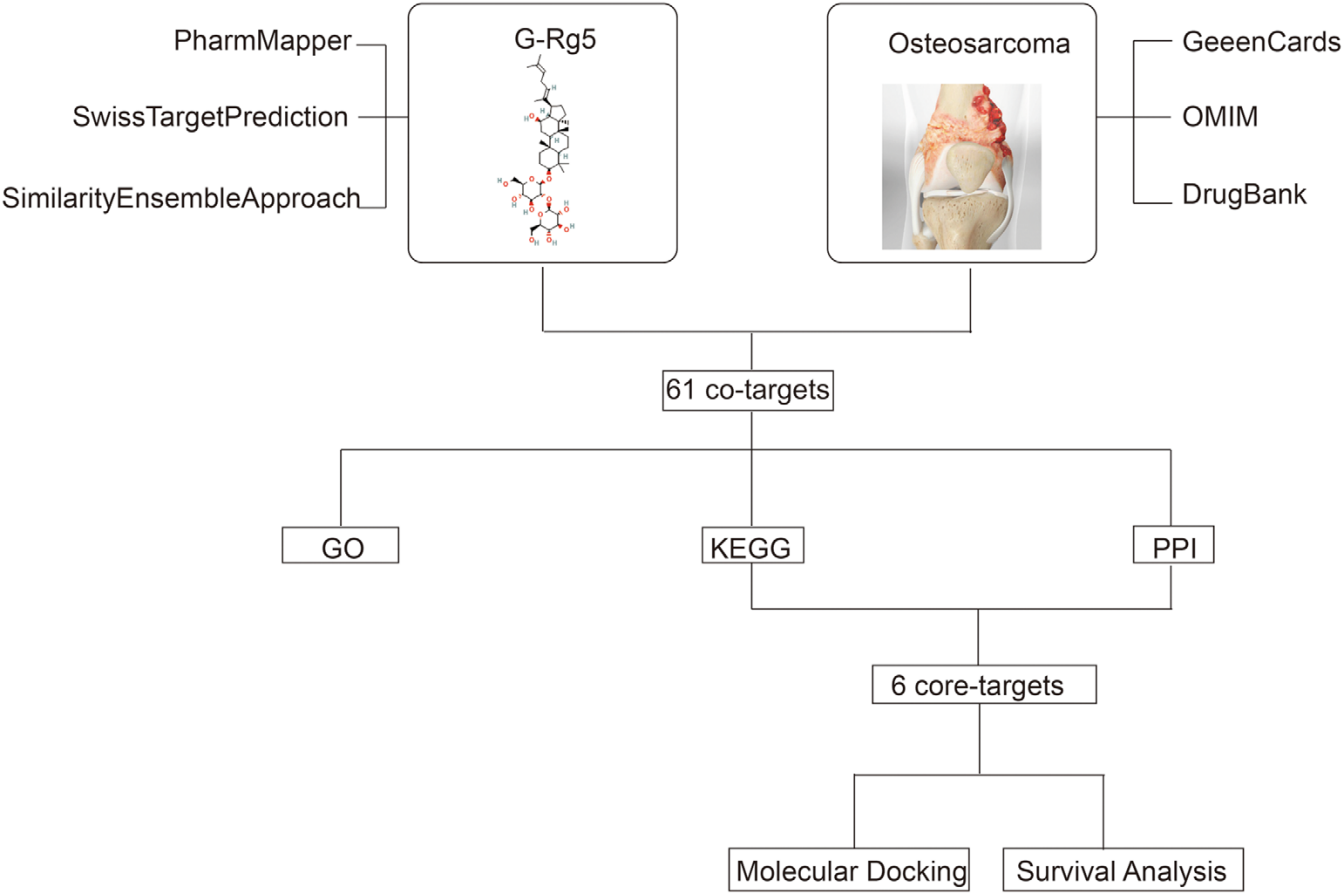
The flow chart of whole analysis for this study.

Natural products extracted from medicinal plants have garnered growing attention because of their biological and clinical therapeutic effects with fewer side effects.^6^, ^7^, ^8^ *Panax ginseng* C.A. Meyer is one of the most widely used herbal medicines in East Asia.^7^ It is reported that *Panax ginseng* C.A. Meyer and its metabolites could induce the apoptosis of tumor cells, inhibit epithelial mesenchymal transition, inhibit angiogenesis, induce cell cycle arrest and reduce multidrug resistance in *in vitro* and *in vivo* tumor models.^9^ The traditional solvent methods for ginsenosides extraction including Soxhlet, heat‐reflux, and shaking techniques. And advanced extraction methods such as ultra‐pressure and ultra‐temperature techniques have gained increasingly attention.^10^ Ginsenoside Rg5 (G‐Rg5, C_42_H_70_O_12_) is a minor ginsenoside extracted from *Panax ginseng* C.A. Meyer with a relative molecular weight of 767.^7^ In a previous study, we found that G‐Rg5 inhibited proliferation and promoted apoptosis of human osteosarcoma cells via PI3K/Akt/mTORC1 mediated LC3 autophagy pathway.^11^ Network pharmacology is a systemic biological method to explore mechanisms of drugs in the treatment of diseases from a comprehensive manner, and provides guidance for drug discovery.^12^, ^13^ The purpose of this study was: (i) to investigate the potential molecular targets and mechanisms by which G‐Rg5 alleviates osteosarcoma through network pharmacology; and (ii) to verify the hub targets of the G‐Rg5 against osteosarcoma through molecular docking and survival analysis. The detailed Flow chart of current study is shown in Figure 1.

## Materials and Methods

### Screening of Potential Targets of G‐Rg5 against Osteosarcoma

Pharmacological targets of G‐Rg5 were obtained from the PharmMapper (http://lilab.ecust.edu.cn/pharmmapper/), SwissTargetPrediction (http://www.swisstargetprediction.ch/) and similarity ensemble approach databases (https://sea.bk slab.org/). In the PharmMapper database, norm fit score was set as greater than 0.7. In the SwissTargetPrediction database, the probability was set as greater than 0. And there was no filter for the similarity ensemble approach database. The targets data obtained from the PharmMapper database were transformed into the corresponding genes through the UniProt database (http:// www.uniprot.org/). The potential target genes of G‐Rg5 were obtained by merging the screening results of the three databases. The key word “osteosarcoma” was searched in the GeneCards (https://www.genecards.org/), Online Mendelian Inheritance in Man (OMIM) (http://www.omim.org), and DrugBank databases (https://go.drugbank.com/). The related genes of osteosarcoma were obtained by merging the screening results of the three databases. The potential targets of G‐Rg5 for the osteosarcoma treatment were obtained by overlapping the target genes of G‐Rg5 and related genes of osteosarcoma, and Venn diagram was drawn using VENNY2.1 (https://bioinfogp.cnb.csic.es/tools/venny/).

### Protein–protein Interaction (PPI) Analysis

The construction of PPI network is very important for the study of protein function. Through PPI analysis, researchers can study on the molecular mechanism of disease systematically and discover new drug targets.^14^ The overlapping targets were imported into the online STRING database, with the species limited to “homo sapiens” and confidence scores > 0.9. And the tsv file of PPI was download after hiding the disconnected nodes, which was imported into Cytoscape 3.8.2 software for analysis and discovering hub target genes. The size and color of nodes were adjusted according to the degree values. Larger and darker nodes mean higher degree values. And the top 10 genes according to the degree values were displayed by using the “count R" and “ggplot” packages of R 4.1.2 software.

### Gene Ontology (GO) and Kyoto Encyclopedia of Genes and Genomes (KEGG) Pathway Analyses

In order to investigate the biological effects and related signaling pathways of the G‐Rg5 against osteosarcoma, the GO function enrichment analysis and KEGG pathway enrichment analysis were performed by the Database for Annotation, Visualization and Integrated Discovery (DAVID) platform. The screening conditions were set as *p* < 0.05 and q < 0.05, and the online bioinformatics analysis tool was used to generate bubble maps (https://www.bioinformatics.com.cn/).Then transporting the related targets of G‐Rg5 for the treatment of osteosarcoma and its corresponding KEGG signaling pathways to the Cytoscape 3.8.2 software to construct a “targets–pathways” network. The size of nodes were adjusted according to the degree values. Larger nodes mean higher degree values. The hub targets were obtained by taking the intersection genes with high degree values in the PPI network and targets‐pathways network.

### Molecular Docking Analysis of Hub Targets

The proteins encoded by hub targets and G‐Rg5 were used as receptors and ligands respectively. The SDF file of a ligand was downloaded from PubChem database, and it was saved as mol2 format after optimized in the ChemBio3D Ultra 14.0 software. The protein structures of receptors were obtained from the PDB database and preprocessed with PyMol software. Using AutoDockTools 1.5.7 software to add polar hydrogens and Kollman charges to receptor and convert ligand and receptor files into pdbqt format, the active pockets of receptors were defined and saved as gpf files. Then AutoDock vina was used for molecular docking. Finally, the docking results were visualized by PyMol software. The binding energy was used to evaluate the binding ability between G‐Rg5 and proteins. It is generally believed that a binding energy less than −7 kcal/mol indicates good binding ability.^10^

### Survival Analysis of Hub Genes

The Kaplan–Meier plotter online database (https://kmplot.com/analysis/) was used to investigate the relationship between the mRNA expression level of hub targets and the 5‐year overall survival (OS) and relapse‐free survival (RFS) rate in sarcoma patients.^3^, ^15^ Hazard ratio (HR) is the ratio of hazards in low gene expression group and high gene expression group. When HR >1, it indicates that the low gene expression increases the risk of a death event; when HR <1, it indicates that the low gene expression reduces the risk of death. A *p* < 0.05 was considered significant difference.

## Results

### Potential Target Genes of G‐Rg5 in the Treatment of Osteosarcoma

**FIGURE 2:**
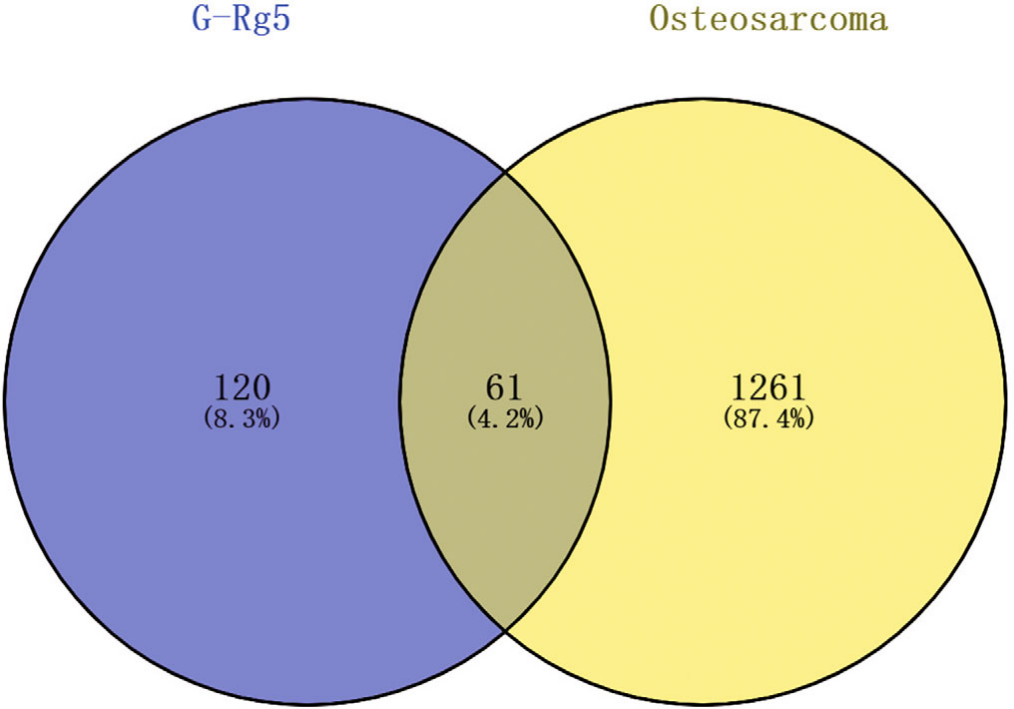
Venn diagram of potential therapeutic targets of ginsenoside Rg5 against osteosarcoma.

A total of 181 pharmacological targets of G‐Rg5 were obtained from the SwissTargetPrediction, similarity ensemble approach and pharmmapper databases; and 1322 related target genes were obtained from the GeneCards, OMIM and DrugBank databases. Through intersection of G‐Rg5 targets and osteosarcoma targets, 61 overlapping target genes were obtained (Table 1, Figure 2).

**TABLE 1:** The information of 61 core targets.

Core targets
BMP2, MMP13, MAPK10, CCNA2, ALB, GSTP1, MTAP, CASP3, PIM1, MAPK1, MMP3, MAPK14, PPARG, FAP, CASP7, SRC, SPARC, KDR, PGR, EGFR, CDK2, ANXA5, MAPK8, CHEK1, PTPN11, ESR1, NR1H2, MIF, DHFR, CTSD, TYMS, AR, BMP7, STAT3, VEGFA, FGF1, FGF2, HSP90AA1, MTOR, MMP9, MAP2K1, MMP1, PIK3CG, ADORA3, MMP2, TOP1, IMPDH2, BCL2L1, ICAM1, MET, MMP14, PLK1, JAK2, CDK4, IL2, DPP4, PIK3CA, ALOX5AP, RB1, TP53, CHEK2

### PPI Network Analysis

**FIGURE 3:**
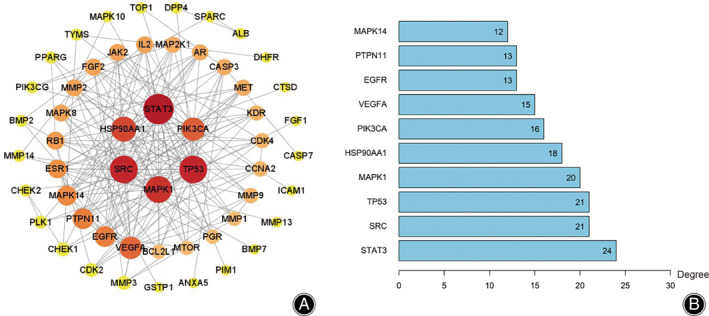
(A) The protein–protein interaction (PPI) network of overlapping targets, (B) The top 10 co‐targets.

The PPI network diagram of the overlapping target genes was obtained by importing the tsv file into Cytoscape 3.8.2 software. According to the degree values of the nodes, the targets were distributed into three concentric circles. The degree values of the outer layer targets were 1 ~ 5, the degree values of the middle layer targets were 6 ~ 15, and the degree values of the inner layer targets were 15–24 (Figure 3A). The bar plot arranged by the degree values of targets was obtained by the “count R” and “ggplot” packages of R language (Figure 3B).

### GO and KEGG Pathway Enrichment Analyses

**FIGURE 4:**
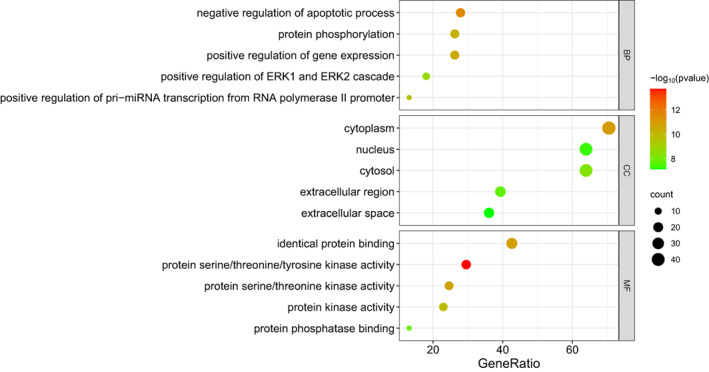
Gene ontology (GO) function enrichment analysis.

**FIGURE 5:**
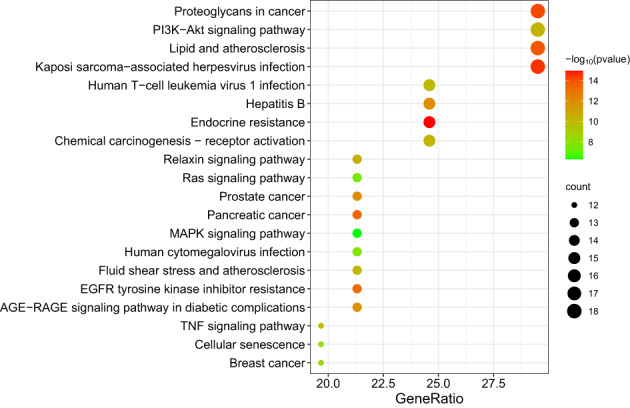
Kyoto Encyclopedia of Genes and Genomes (KEGG) pathway enrichment analysis.

**FIGURE 6:**
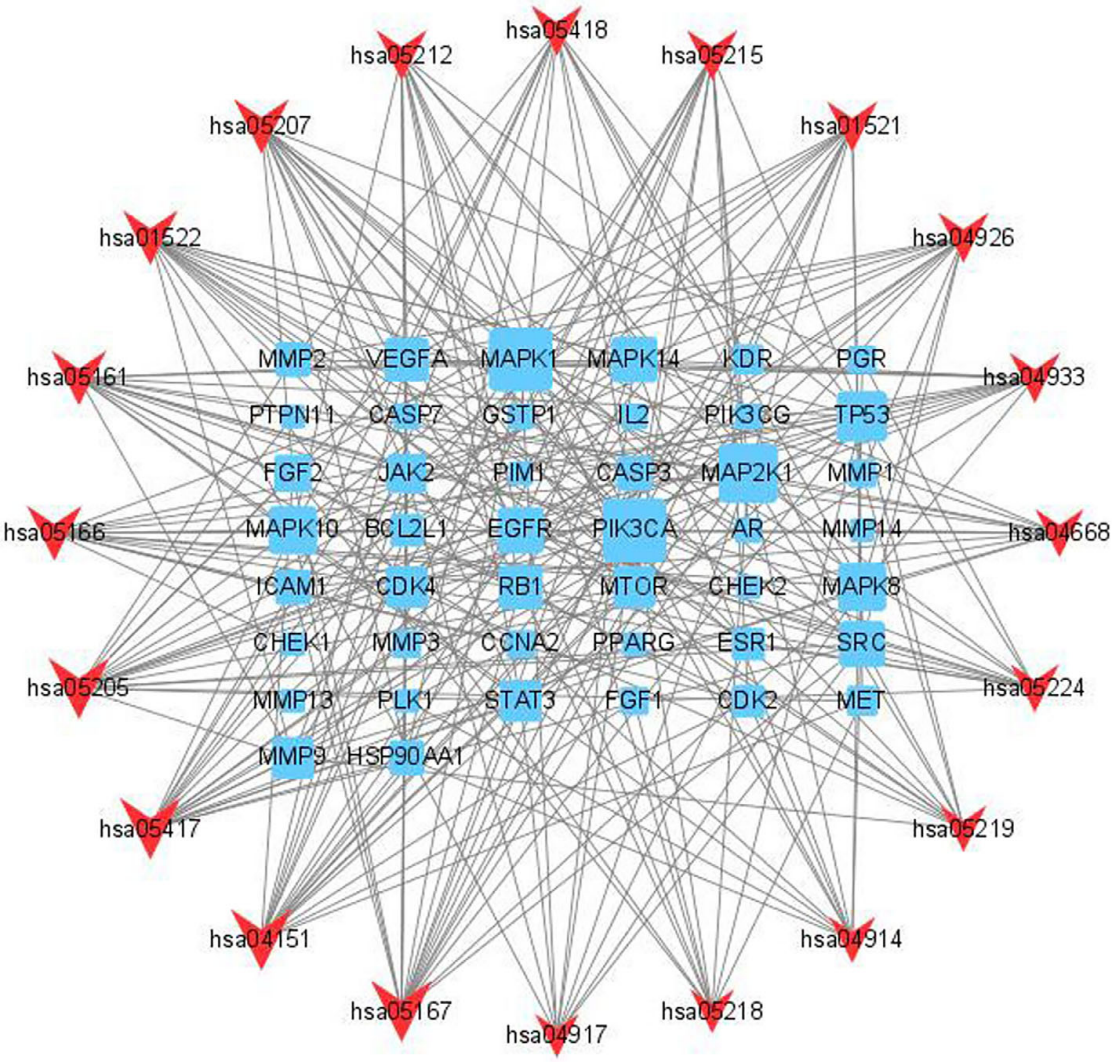
Targets–pathways network. Rectangles represent targets, and v shapes represent pathways.

The top five entries for BP included negative regulation of the apoptotic process, protein phosphorylation, positive regulation of gene expression, positive regulation of ERK1 and ERK2 cascades, and positive regulation of pri − miRNA transcription from the RNA polymerase II promoter. The top five entries for CC included cytoplasm, nucleus, cytosol, extracellular region and extracellular space. The top five entries for MF included identical protein binding, protein serine/threonine/tyrosine kinase activity, protein serine/threonine kinase activity, protein kinase activity and protein phosphatase binding (Figure 4). The top ten signaling pathways included Proteoglycans in cancer (hsa05205), PI3K − Akt signaling pathway (hsa04151), lipid and atherosclerosis (hsa05417), Kaposi sarcoma−associated herpesvirus infection (hsa05167), human T − cell leukemia virus 1 infection (hsa05166), hepatitis B (hsa05161), endocrine resistance (hsa01522), chemical carcinogenesis‐receptor activation (hsa05207), relaxin signaling pathway (hsa04926) and Ras signaling pathway (hsa04014) (Figure 5). The targets‐pathways network containing 64 nodes and 276 edges was constructed with the top 20 pathways and 44 target genes by Cytoscape 3.8.2, in which 11 genes had high degree values (degree values ≥ 10), including PIK3CA (degree value = 19), MAPK1 (degree value = 19), MAP2K1 (degree value = 17), TP53 (degree value = 13), MAPK8 (degree value = 12), MAPK10 (degree value = 12), EGFR (degree value = 11), SRC (degree value = 11), MAPK14 (degree value = 11), VEGFA (degree value = 10) and RB1 (degree value = 10) (Figure 6).

### Molecular Docking Results

**FIGURE 7:**
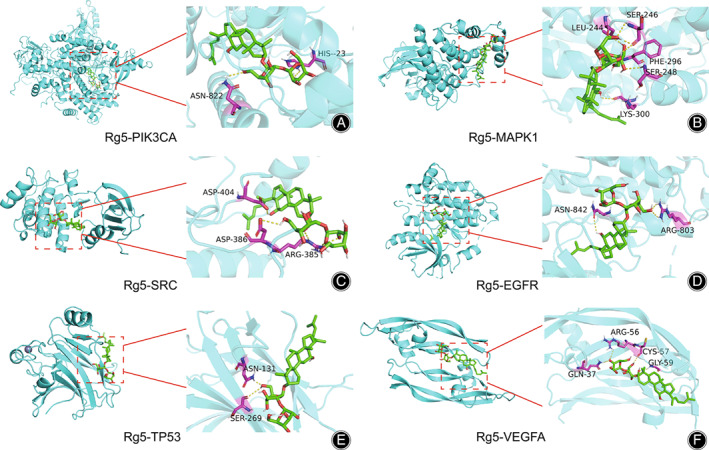
Schematic diagram of the docking results: (A) PIK3CA and ginsenoside Rg5, (B) MAPK1 and gisneoside Rg5, (C) SRC and gisenoside Rg5, (D) EGFR and gisenoside Rg5, (E) TP53 and gisenoside Rg5, (F) VEGFA and gisenoside Rg5.

Six hub targets including PIK3CA (PBD ID: 2RD0), SRC (PDB ID: 3F3V), TP53 (PBD ID: 1TSR), MAPK1 (PBD ID: 5NHV), EGFR (PBD ID: 1XKK) and VEGFA (1VPF) were chosen according to PPI and targets–pathways network analyses and then performed molecular docking with G‐Rg5. The results showed that the binding energy of G‐Rg5 to PIK3CA was −10.7 kcal/mol, to SRC was −7.6 kcal/mol, to TP53 was −7.6 kcal/mol, to MAPK1 was −9.0 kcal/mol, to EGFR was −8.8 kcal/mol and to VEGFA was −8.4 kcal/mol. The results of molecular docking showed that G‐Rg5 had strong affinity with hub targets (Figure 7).

### Survival Analysis Results

**FIGURE 8:**
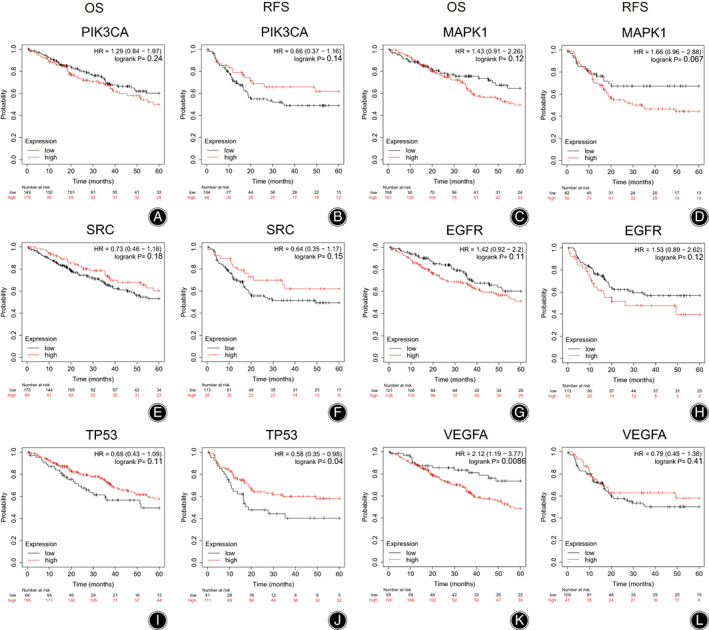
Survival analysis of hub genes: (A) 5‐year OS for PIK3CA, (B) 5‐year RFS for PIK3CA, (C) 5‐year OS for MAPK1, (D) 5‐year RFS for MAPK1, (E) 5‐year OS for SRC, (F) 5‐year RFS for SRC, (G) 5‐year OS for EGFR, (H) 5‐year RFS for EGFR, (I) 5‐year OS for TP53, (J) 5‐year RFS for TP53, (K) 5‐year OS for VEGFA, (L) 5‐year RFS for VEGFA. Red curve represents high expression group, and black curve represents low expression group.

Correlation analysis showed that sarcoma patients with high VEGFA expression showed a poor 5‐year OS than that with low VEGFA expression (*p* = 0.0086), and low TP53 expression showed a poor 5‐year RFS than that with high TP53 expression (*p* = 0.04). Other genes were not significantly correlated with the prognosis in 5‐year OS and RFS (all *p*‐values > 0.05) (Figure 8).

## Discussion

### Main Findings

The current research investigated the mechanism of G‐Rg5 for the treatment of osteosarcoma by network pharmacology and molecular docking. Through database search, 61 overlapping target genes were obtained by intersecting the targets of G‐Rg5 and osteosarcoma. The results of KEGG analysis revealed that the main related signaling pathways including PI3K‐Akt signaling pathway, Proteoglycans in cancer, lipid and atherosclerosis and Kaposi sarcoma‐associated herpesvirus infection. PIK3CA, SRC, TP53, MAPK1, EGFR, and VEGFA were selected by the PPI and targets–pathways networks analyses for further molecular docking and survival analysis. The results of molecular docking showed that the binding energies between G‐Rg5 and hub targets were all stable. And survival analysis showed TP53 and VEGFA were correlated with the prognosis of sarcoma patients.

### Potential Signaling Pathways of G‐Rg5 against Osteosarcoma

The PI3K/Akt signaling pathway is considered to be one of the crucial oncogenic pathways in the development of osteosarcoma including apoptosis inhibition, proliferation, invasion, angiogenesis, metastasis and chemoresistance.^16^ Down‐regulation of this signaling pathway by small molecule compounds appears to be an attractive potential approach for osteosarcoma treatment.^16^ For instance, Li *et al*.^17^ reported that ginsenoside Rg3 could suppress proliferation, migration and induce apoptosis of human osteosarcoma cells *via* inhibiting the PI3K/Akt signaling pathway. In a previous study, we found that G‐Rg5 inhibited proliferation and induced apoptosis of human osteosarcoma cells through activating autophagy by inhibiting PI3K/Akt/mTORC1 signaling pathway, which is consistent with the results of current research.^11^ Proteoglycans are important components of the extracellular matrix which can be used as a prognostic biomarker and potential therapeutic targets for osteosarcoma.^18^, ^19^ Another signaling pathway of Kaposi sarcoma−associated herpesvirus infection has also been demonstrated to be associated with osteosarcoma. Chen *et al*.^20^ found that the seroprevalence of Kaposi's sarcoma‐associated herpesvirus in osteosarcoma patients was significantly higher than normal controls. Furthermore, gene expression profile analysis of osteosarcoma samples demonstrated that Kaposi's sarcoma‐associated herpesvirus infection regulated the genes and signaling pathways associated with the progression of osteosarcoma, which indicated a close relation between Kaposi's sarcoma‐associated herpesvirus and osteosarcoma.^20^

### Potential Hub Genes of G‐Rg5 against Osteosarcoma

The hub targets obtained by PPI network and KEGG pathway enrichment analyses include PIK3CA, TP53, SRC, EGFR, MAPK1, and VEGFA. The molecular docking revealed the binding between G‐Rg5 and PIK3CA exhibited the best binding energy (−10.7 kJ/mol). The protein encoded by PIK3CA represents the p110α catalytic subunit of PI3K and displays a serine‐protein kinase activity, which is the core component of PI3K/Akt signaling pathway.^21^ Qu *et al*.^22^ reported that down‐regulation of PRKCI activated osteosarcoma cells autophagy *via* inhibiting the PIK3CA/Akt/mTOR signaling pathway. We speculated that G‐Rg5 could exert anti‐osteosarcoma effects by binding to the p110α catalytic subunit of PI3K and contribute to the regulation of downstream targets that involve in PI3K/Akt signaling pathway. The results of the survival analysis revealed that VEGFA as a risk factor in osteosarcoma treatment. VEGFA is a member of the PDGF/VEGF family. The protein encoded by VEGFA can induce migration and invasion of vascular endothelial cells, which is crucial for tumor angiogenesis and metastasis.^23^ High VEGFA expression indicates unfavorable prognosis in osteosarcoma patients.^24^ Vimalraj *et al*.^25^ reported that melatonin suppressed osteosarcoma angiogenesis *via* targeting VEGFA. Another study showed that MicroRNA‐134 attenuated osteosarcoma angiogenesis and proliferation *via* inhibiting the VEGFA/VEGFR1 signaling pathway.^26^ In the current study, G‐Rg5 showed strong binding energy with VEGFA (−8.4 kcal/mol), which suggested that G‐Rg5 could potentially inhibit the activity of VEGFA and VEGFA‐related pathways, and therefore suppress osteosarcoma growth and metastasis. We also found that TP53 as a protective factor by survival analysis. P53 encoded by the TP53 gene acted as a tumor suppressor in the human body, and TP53 mutant has been reported to be associated with promoted malignancy and poor prognosis of osteosarcoma.^27^ Therefore, targeting TP53 could be a promising option in the osteosarcoma treatment. In addition, the protein encoded by SRC is a non‐receptor tyrosine kinase protein that involves in various intracellular signaling transduction related to osteosarcoma. For instance, inhibition of the SRC/STAT3 signaling pathway by ameloblastin was shown to induce apoptosis and suppress chemoresistance of osteosarcoma cells.^28^ And migration and invasion of human osteosarcoma cells could be inhibited by targeting SRC.^29^ A recent study demonstrated that bavachin promoted ferroptosis of osteosarcoma cells through the STAT3/P53/SLC7A11 axis.^30^ MAPK1 is a downstream oncogenic gene of the MAPK signaling pathway, and down‐regulation of MAPK1 by MicroRNA‐511 can inhibit the proliferation of osteosarcoma cells and osteosarcoma metastasis.^31^

### Limitations and Prospect

The current study was based on *in silico* analyses, which should be further validated by *in vitro* and *in vivo* experiments. For instance, there are significant differences between different subtypes of osteosarcoma. However, the survival analysis in the current study is through an online database, and it is unable to perform survival analysis differentiated by subtypes, and the effects of G‐Rg5 on osteosarcoma based on clinical samples should be further investigated in the future. In addition, considering G‐Rg5 had strong binding capacity with VEGFA by molecular docking, the anti‐angiogenesis and anti‐metastasis effects of G‐Rg5 on osteosarcoma could be another research direction.

## Conclusion

This study analyzed the relationship between the pharmacological targets of G‐Rg5 and the related genes of osteosarcoma by network pharmacology method. We predicted that G‐Rg5 has the characteristics of multiple‐targets and multi‐pathways in the treatment of osteosarcoma, which are closely related to promoting apoptosis, regulating autophagy, anti‐proliferation, anti‐metastasis, anti‐angiogenesis, and anti‐chemoresistance of tumor cells. PIK3CA, SRC, TP53, MAPK1, EGFR, and VEGFA are potential hub targets. This study provides new directions for experimental and clinical research on the therapeutic effects of G‐Rg5 against osteosarcoma.

## Author Contributions

Concept: Ming‐yang Liu and Yan‐zheng Gao. Methodology: Ming‐Yang Liu and Xiang Zhao. Software: Zhen‐dong Liu, Liang Zhang and Yu Zhang. Writing: Ming‐Yang Liu and Dong‐xin Jiang. Tables and figures: Run‐ze Liu and Xiao‐yu Rong. Suggestions: Hai‐jun Li.

## Funding Information

Henan Postdoctoral Fund (Grant ID: 202103115), Henan Provincial Medical Science and Technology Tackling Program Provincial‐Ministerial Co‐construction Project (SBGJ202103019).

## Disclosure Statement

The authors declare that they have no conflicts of interest.

## Ethics Statement

Not applicable.

## Data Availability

The datasets used during the current study are available from the corresponding author on reasonable request.
